# The impact of METTL3 on bladder cancer through m^6^A modification: a potential therapeutic target and prognostic biomarker

**DOI:** 10.3389/fonc.2025.1622117

**Published:** 2025-07-03

**Authors:** Dong Wen, Pengcheng Fu, Geliu Shuai, Yuebin Wang, Shengxin Yu, Huiquan Liu, Wei Wan, Junrong Zou, Xiaofeng Zou

**Affiliations:** ^1^ The First Clinical Medical College, Gannan Medical University, Ganzhou, Jiangxi, China; ^2^ Department of Urology, The First Affiliated Hospital of Gannan Medical University, Ganzhou, Jiangxi, China; ^3^ Institute of Urology, The First Affiliated Hospital of Gannan Medical University, Ganzhou, Jiangxi, China

**Keywords:** m^6^A, METTL3, bladder cancer, bladder cancer treatment, bladder cancer drug resistance, bladder cancer prognosis

## Abstract

Bladder cancer (BCa) is one of the most prevalent malignant tumors globally, particularly among men. According to data from the Global Cancer Research Agency, the annual incidence of BCa continues to rise, and its clinical features are complex, involving various molecular mechanisms and pathophysiological processes. Although existing treatments such as surgery, chemotherapy, and immunotherapy have improved patient prognosis to some extent, many individuals remain at risk for recurrence and metastasis. Therefore, there is an urgent need to explore new biomarkers and therapeutic targets to enhance the diagnostic and therapeutic efficacy of BCa. In recent years, RNA methylation, as an important post-transcriptional modification, has gradually attracted the attention of researchers. Among the methyltransferases, methyltransferase-like 3 (METTL3) is considered a key regulator, which is mainly responsible for the N6-methyladenosine (m^6^A) modification of mRNA. More and more studies have shown that METTL3 not only plays an important role in normal physiological processes, but also is closely related to the occurrence and development of a variety of tumors. This review aims to systematically explore the role of METTL3 in BCa, including its biological function, expression characteristics, potential therapeutic targets, and prognosis related research progress. Through the in-depth analysis of METTL3, we hope to provide new ideas and directions for the early diagnosis, prognostic evaluation, and the development of novel treatment strategies for BCa.

## 1 Introduction

BCa is one of the most prevalent malignant tumors of the urinary system. In terms of incidence, BCa ranks as the 10th most common cancer worldwide, with an estimated 600,000 new cases reported in 2022 (1). In recent years, both the incidence and mortality rates of BCa in China have been on the rise. Urothelial carcinoma (UBC) is the most common histological subtype, accounting for approximately 90% of BCa cases globally (2). UBC is typically classified into two categories: non-muscle invasive bladder Cancer (NMIBC) and muscle invasive bladder cancer (MIBC). At the time of diagnosis, 75% of UBC cases are classified as NMIBC, while 25% are categorized as MIBC or metastatic disease. For patients with NMIBC, a common treatment strategy involves transurethral bladder tumor resection followed by postoperative intravesical chemotherapy or Bacillus Calmette-Guérin (BCG) therapy (3, 4). For patients with locally advanced or advanced MIBC, the standard treatment remains the gemcitabine and cisplatin (GC) regimen (5). However, once MIBC has metastasized, the five-year survival rate drops to only 15% (6). BCa not only brings physical pain to patients, but also suffers from anxiety, fear and torture psychologically. At the same time, families have to bear financial pressure and the rhythm of life is disrupted. Therefore, there is an urgent need to explore new treatment strategies and personalized treatment methods (7). As an RNA methyltransferase, METTL3 has attracted much attention in cancer research in recent years. Studies have shown that METTL3 regulates gene expression and cell fate by adding m^6^A modification to mRNA (8), and affects biological processes such as cell proliferation, apoptosis and migration (9). The expression level of METTL3 is abnormally increased in a variety of tumor types, such as lung, liver, breast, gastric, colorectal and pancreatic cancer (10–15), and is closely related to the occurrence, development and prognosis of tumors. In BCa, high expression of METTL3 is thought to be associated with tumor aggressiveness and metastasis (16). METTL3 can participate in the biological behavior of BCa by regulating key genes related to cell cycle, apoptosis and chemotherapy resistance (17). In addition, the expression level of METTL3 may also be used as a prognostic marker in patients with BCa (18), providing a new risk assessment tool for clinical practice. This review focuses on the potential of METTL3 as a potential therapeutic target as well as a prognostic marker in the treatment of BCa by promoting the proliferation and invasion of BCa cells.

## 2 M^6^A methylation modification

To date, more than 170 types of post-transcriptional RNA modifications have been identified (19), with m^6^A being the most prevalent RNA modification found in eukaryotic mRNAs. Numerous studies have demonstrated that m^6^A modifications play a crucial role in regulating RNA processing, splicing, nucleation, translation, and stability. These modifications significantly impact human diseases, as alterations in m^6^A may promote tumor development (20, 21) or contribute to neurodegeneration (22–24). In addition to its role in mRNA, m^6^A modification is also present in non-coding RNAs, such as microRNAs(miRNAs), long non-coding RNAs(lncRNAs), and circular RNAs(circRNAs), which similarly regulate their biological functions (25–27). m^6^A modulates gene expression in a post-transcriptional manner, involving three parts: “writers,” (28–30) “erasers,” (31, 32) and “readers” (33–35) (see **Table 1**). Following modification by these three components, the primary transcript RNA is transformed into mature RNA.

**Table 1:** m^6^A post-transcriptional regulation mechanisms: writers, erasers, and readers.

Functional module	Key constituent	Key Features	References
Writers	METTL3, METTL14, WTAP, VIRMA, RBM15/15B	Adds m^6^A modifications to RNA, regulating RNA stability and translation efficiency. It is also involved in RNA splicing, nuclear export, and localization.	(28–30)
Erasers	FTO, ALKBH5	Dynamically removes m^6^A modifications, regulates RNA stability and translation, and influences the development of diseases such as obesity, cancer, and neurodegenerative disorders.	(31, 32)
Readers	YTH Family (YTHDF1, YTHDF2, YTHDF3),YTHDC Family (YTHDC1, YTHDC2), IGF2BP Family:IGF2BP1, IGF2BP2, IGF2BP3), hnRNP Family: (hnRNPC, hnRNPG)	Recognizes and binds to m^6^A modification sites, determining the fate of RNA—whether it is translated, stabilized, degraded, or spliced—and coordinates the role of RNAs in various biological environments.	(33–35)

METTL3,methyltransferase-like 3; METTL14,methyltransferase 14; WTAP, wilms tumor 1-associated protein; VIRMA, viral RNA methylation adapter; RBM15/15B,RNA binding motif protein 15/15B; FTO, fat mass and obesity-associated protein; ALKBH5,alkB homolog 5; YTHDF1/2/3, YTH N6-methyladenosine RNA binding protein 1/2/3; IGF2BP1/2/3, Insulin like growth factor 2 MRNA binding protein 1/2/3.

## 3 Structure and function of METTL3

METTL3, also known as MT-A70, is a 70 kDa protein that serves as a key m^6^A methyltransferase and is widely present in eukaryotes (36). The structural features of METTL3 include an S-adenosylmethionine (SAM) binding domain and an RNA-binding domain. The N-terminus of METTL3 contains two Cys-Cys-Cys-His (CCCH)-type zinc finger (ZnF) motifs, which are commonly found in RNA-binding proteins (37). These structures enable METTL3 to efficiently catalyze methyl transfer reactions. Typically, METTL3 forms a stable dimeric complex with methyltransferase 14 (METTL14) in the nucleus, which subsequently interacts with wilms’ tumor 1-associating protein (WTAP) to create the m^6^A methyltransferase complex (METTL3/METTL14/WTAP), also referred to as m^6^A “writers” (38). “In the methyltransferase complex, METTL3 is the first and only catalytic subunit discovered to transfer methyl groups from SAM to adenosine residues in the RNA molecule, forming the m^6^A modification (39).” This process is an important part of RNA post-transcriptional modification, affecting mRNA stability and translational efficiency. In addition, METTL3 also plays an important role in biological processes such as stem cell differentiation (40), immune cell activation (41), and neural development (42), helping cells to respond to different environments. In terms of disease, the abnormal expression of METTL3 is closely related to the occurrence and development of a variety of cancers, and may affect the behavior of tumors by regulating the proliferation, metastasis and drug resistance of tumor cells. Studies have found that microRNA-600 (miR-600) can inhibit the progression of lung cancer by down-regulating the expression of METTL3 (10). He et al. found that microRNA-4429 (miR-4429) can inhibit m^6^A modification by targeting METTL3, leading to the stabilization of SEC62 homolog, preprotein translocation factor (SEC62) to prevent the progression of gastric cancer (13). Interestingly, METTL3 is also closely related to neurodegenerative diseases. It has methyltransferase activity and deposits methyl groups on RNA, which can inactivate neurophysiological events and trigger or worsen neuropathological events (23). Overall, the structure and function of METTL3 complement each other, making it an important factor in the regulation of m^6^A modification.

## 4 The role of m^6^A modification and its key proteins in BCa.

Studies have demonstrated that m^6^A modification is closely associated with the occurrence, progression, and prognosis of BCa. Compared to normal bladder tissue, the expression patterns of various regulatory factors involved in m^6^A modification differ significantly. Several key regulators are notably upregulated in BCa cells, including METTL3, WTAP (43), fat mass and obesity-associated protein (FTO) (44), insulin-like growth factor 2 mRNA-binding protein 1 (IGF2BP1) (45), YTH n6-methyladenosine RNA-binding protein 1 (YTHDF1) (46), and Heterogeneous nuclear ribonucleoprotein A2/B1 (HNRNPA2B1) (47). Conversely, AlkB homolog 5 (ALKBH5) (48), METTL14 (49), and YTH n6-methyladenosine RNA-binding protein 3 (YTHDF3) (50) are downregulated. FTO is recognized as an oncogenic factor in the development of BCa. The knockdown of FTO enhances the stability of the mRNA for the signal transducer and activator of transcription 3 (STAT3), increases STAT3 expression, effectively reduces cell cycle progression, and diminishes cell proliferation, migration, and invasion capabilities, while also inducing apoptosis and carcinogenic transformation. An increase in FTO levels correlates with poor prognosis in BCa patients (51, 52). Qiu et al. found that knockdown of yes-associated protein 1(YAP1) inhibited the growth, invasion, and migration of BCa cells, and at the same time, hindered YTHDF3-mediated degradation of SMAD family member 7 (SMAD7), ultimately leading to a reduction in the stemness of BCa cells (50). YTHDF1 plays a role in BCa progression and glycolytic activity. It has been found that YTHDF1 can positively regulate the expression of glutamate ionotropic receptor NMDA type subunit 2D (GRIN2D) to promote BCa cell proliferation and enhance aerobic glycolysis. Moreover, inhibition of the m^6^A-YTHDF1-GRIN2D axis can inhibit cancer progression and metabolic changes (53). Huang et al. found that overexpression of METTL14 inhibited BCa cell migration, invasion *in vitro*, and tumor metastasis *in vivo*. METTL14 positively regulated ubiquitin-specific peptidase 38 (USP38) and enhanced the stability of USP38 mRNA through YTHDF2-dependent m^6^A modification. To inhibit migration, invasion, and epithelial-mesenchymal transition (EMT) of BCa cells (54). In addition, both YTHDC1 and ALKBH5 mediate cisplatin resistance in BCa. YTHDC1 could decrease phosphatase and tensin homolog (PTEN) expression and activate PI3K/AKT signaling by destabilizing PTEN mRNA while enhancing cell viability in BCa cells.” Thus, reduction of YTHDC1 expression promotes resistance to cisplatin, whereas overexpression of YTHDC1 promotes cisplatin sensitivity (55). “ Meanwhile, it has been reported that ALKBH5 knockdown can also promote the proliferation, migration and invasion of BCa cells and sensitize BCa cells to cisplatin *in vitro* and *in vivo* in an m^6^A-dependent manner through the casein kinase 2 (CK2)-mediated glycolysis pathway (56). In summary, m^6^A modification plays a multifaceted role in the occurrence and development of BCa, affecting the proliferation, migration, invasion and cisplatin resistance of tumor cells by dynamically regulating the fate of RNA. Although current studies on m^6^A modification in BCa have revealed remarkable functions, many questions remain unanswered.

## 5 The role of METTL3 in BCa

**Figure 1:**
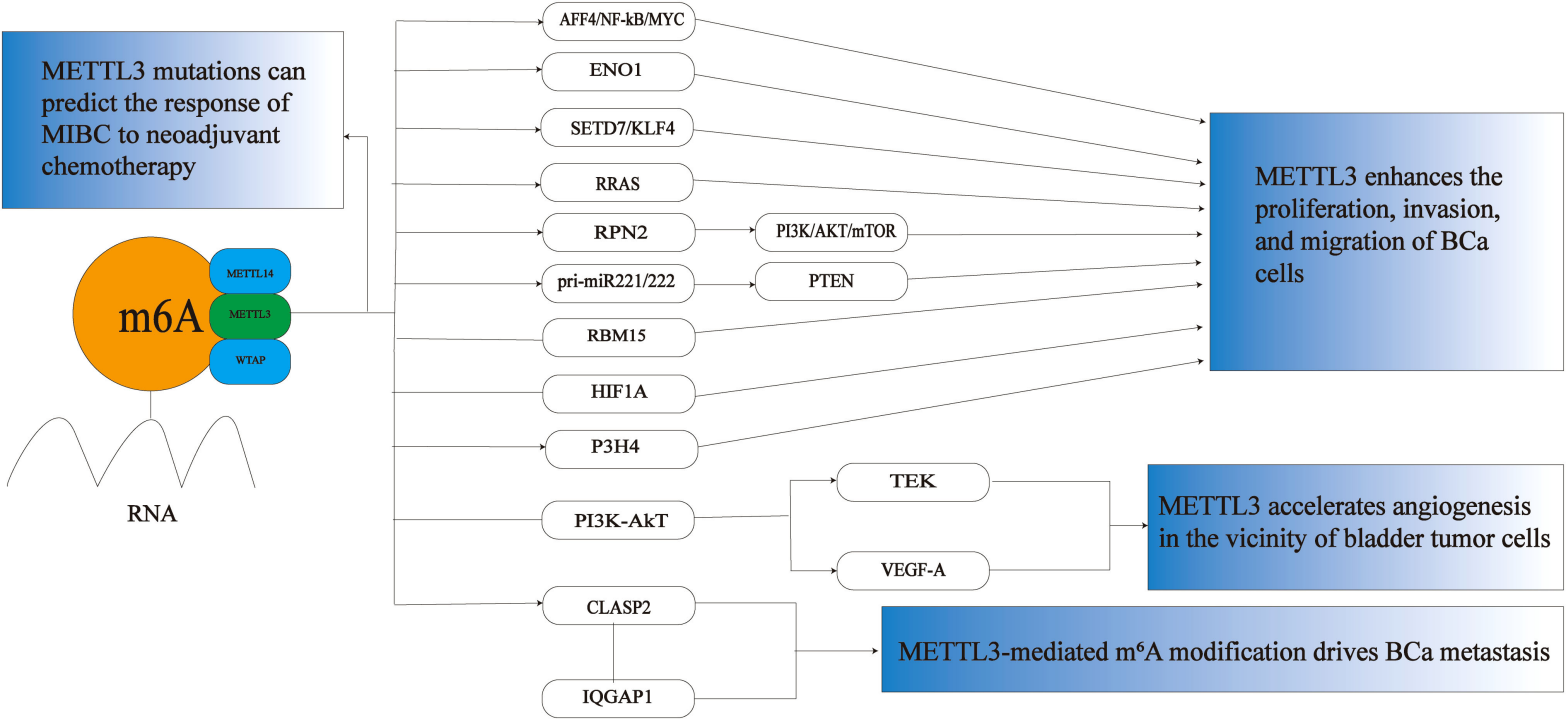
The role of METTL3 in BCa. METTL3 plays a crucial role in BCa primarily by promoting the proliferation, invasion, and migration of BCa cells. It accelerates angiogenesis around bladder tumor cells, drives the metastasis of BCa, and can also predict the response of MIBC to neoadjuvant chemotherapy. M^6^A,N6-methyladenosine; MIBC, muscle invasive bladder cancer; BCa, bladder cancer; AFF4/NF-κB/MYC,FMR2 family member 4/nuclear factor kappa-light-chain-enhancer of activated B cells/MYC proto-oncogene;ENO1,Enolase 1,SETD7,SET domain containing 7; KLF4,kruppel-like factor 4; RRAS,RAS-related protein R-Ras; RPN2,ribophorin II; pri-miR-221/222,Primary microRNA-221/222;RBM15,RNA binding motif protein 15; HIF1A,hypoxia-inducible factor 1 alpha; P3H4,prolyl 3-hydroxylase 4; PI3K/Akt, phosphoinositide 3-kinase/protein kinase B;TEK,TEK tyrosine kinase; VEGF-A, vascular endothelial growth factor A;CLASP2,Cytoplasmic linker associated protein 2; IQGAP1,IQ motif containing GTPase activating protein 1.

As one of the key methyltransferases involved in m^6^A methylation modification, METTL3 plays a significant role in the occurrence and progression of BCa (see **Figure 1**). Studies have demonstrated that METTL3 precisely regulates the expression of tumor-related genes by modulating the levels of m^6^A modification. This regulation promotes the proliferation, invasion, and migration of tumor cells and may also contribute to the remodeling of the tumor microenvironment and the development of chemotherapy resistance. Its abnormally high expression is often closely associated with poor prognosis in patients with BCa, highlighting its considerable potential for use in diagnosis, prognosis assessment, and targeted therapy.

### 5.1 METTL3 enhances the proliferation, invasion, and migration of BCa cells

More and more studies have found that METTL3 shows a significant upward trend in BCa tumor tissues, and the overexpression of METTL3 significantly promotes the growth and invasion of BCa cells. METTL3 can promote cancer cell proliferation and anti-apoptosis by regulating various targets and pathways, including miRNAs and non-coding RNAs, which are critical for BCa. Cheng et al. first revealed METTL3-mediated m^6^A modification in BCa cells. Knockdown of METTL3 significantly reduced the proliferation, invasion, *in vitro* survival rate, and *in vivo* tumorigenicity of BCa cells. At the same time, FMR2 family member 4/nuclear factor kappa-light-chain-enhancer of activated B cells/MYC proto-oncogene (AFF4/NF-κB/MYC) was further identified as the direct targets of METTL3. It can promote the progression of BCa through this signaling pathway (57). In addition, Shen et al. found that oncogene enolase 1 (ENO1) was methylated and highly expressed in BCa, and the RNA binding motif protein 15 (RBM15)/METTL3 complex enhanced the translation efficiency of ENO1 mRNA through m^6^A modification, thereby promoting BCa cell proliferation (58). YTHDF2, the first discovered m^6^A “reader” protein, regulates mRNA degradation and cell viability (59, 60). Mettl3-mediated m^6^A modification is recognized by YTHDF2, which mediates the mRNA reduction of tumor suppressors SET domain containing 7 (SETD7) and kruppel-like factor 4 (KLF4). In turn, it induces the progression of BCa (61). In addition, YTHDF2 may also bind to the m^6^A modification site RAS-related protein R-Ras (RRAS) and cause the degradation of RRAS mRNA, and bioinformatics analysis showed that RRAS is a potential downstream target of METTL3. METTL3 can bind to the m^6^A site of RRAS mRNA and inhibit the transcriptional activity of RRAS, thereby promoting the proliferation, migration, and invasion of BCa cells (62). Studies have found that METTL3 can also bind to YTHDF1 to significantly reduce the ribophorin II (RPN2) mRNA and protein, thereby reducing the phosphorylation level of the phosphoinositide 3-kinase/protein kinase B/mammalian target of rapamycin (PI3K/AKT/mTOR). pathway and leading to proliferation of BCa cells (63). Recent studies have found that METTL3 can interact with microprocessor protein DiGeorge syndrome critical region gene 8 (DGCR8) and positively regulate and accelerate the maturation of Primary microRNA-221/222 (pri-miR-221/222) in an m^6^A-dependent manner, leading to PTEN reduction and ultimately promoting the proliferation of BCa (64). Huang et al. found that RNA binding motif protein 15(RBM15) and METTL3 are potential master regulators of lncRNAs, and the level of m^6^A modification of lncRNA was significantly reduced after knocking down METTL3 and RBM15. This suggests that METTL3 and RBM15 may promote the development and progression of BCa by jointly regulating the level of RNA modification (16). Interestingly, Liu et al. found that long-term exposure to fine particulate matter is also closely related to the development of BCa, and particulate matter 2.5 (PM2.5) can enhance the expression of METTL3 by inducing hypomethylation of its promoter and increasing the binding affinity of transcription factor hypoxia-inducible factor 1 alpha (HIF1A). PM2.5 exposure exerts epigenetic regulation on BCa through the HIF1A/METTL3 network (65). EMT is an important biological process in the development of cancer and plays a key role in the invasion and metastasis of tumor cells (66). Liu et al. found that prolyl 3-hydroxylase 4 (P3H4) was significantly highly expressed in BCa samples, and METTL3 overexpression increased the stability of P3H4 mRNA, thereby promoting proliferation, migration, invasion, and EMT progression in BCa (67). Taken together, these studies suggest that METTL3 acts as an oncogene contributing to BCa progression and metastasis.

### 5.2 METTL3 accelerates angiogenesis in the vicinity of bladder tumor cells

Tumor blood vessel formation plays a crucial role in the growth of primary solid tumors, as well as in tumor progression and metastasis. An increase in tumor blood vessel density facilitates the entry of tumor cells into the circulation, and this enhanced blood vessel formation appears to be closely associated with the invasive characteristics of tumors (68). Vascular endothelial growth factor (VEGF), also known as vascular permeability factor (VPF) (69), has been identified as a significant pro-angiogenic factor that is frequently overexpressed in various tumors, including BCa, breast cancer (70). VEGF can induce the proliferation, migration, and neovascularization of vascular endothelial cells in the normal tissues surrounding BCa, thereby providing adequate oxygen and nutrients to the tumor (71). Targeting VEGF, its receptors, and downstream signaling cascades represents a viable strategy to inhibit BCa growth and metastasis (72). Wang et al. found that METTL3 could promote angiogenesis, epithelial-mesenchymal transition, and metastasis in BCa by regulating the phosphoinositide 3-kinase/protein kinase B (PI3K/AkT) signaling pathway, and METTL3 inhibited transcripts and proteins of TEK tyrosine kinase (TEK) and vascular endothelial growth factor A (VEGF-A) involved in the PI3K/AKT pathway. In addition, in order to further study whether METTL3-mediated m^6^A modification could effectively affect the biological process of BCa, gene ontology (GO) analysis of the m^6^A-enriched gene set showed that METTL3 methylation could accelerate the formation of neovascularization around BCa and promote the progression of BCa (73).

### 5.3 METTL3-mediated m^6^A modification drives BCa metastasis

Metastasis of BCa is a complex, multistep process primarily triggered by cytoskeletal reorganization. Cytoplasmic linker associated protein 2 (CLASP2), a microtubule-binding protein, plays a crucial role in the dynamic regulation of the cytoskeleton and cell migration (74). Elevated expression of CLASP2 is associated with shorter overall survival in BCa patients (75). Tumor necrosis factor-alpha (TNF-α) has been shown to promote METTL3-mediated m^6^A modification of CLASP2, thereby enhancing the stability of CLASP2 mRNA. Additionally, CLASP2 interacts with IQ motif-containing IQ motif containing GTPase activating protein 1 (IQGAP1). Consequently, the remodeling of the F-actin cytoskeleton drives the metastasis of BCa (76).

### 5.4 METTL3 mutations can predict the response of MIBC to neoadjuvant chemotherapy

MIBC is a highly aggressive subtype of BCa, characterized by tumor invasion into the muscular layer of the bladder. This subtype is associated with a higher risk of metastasis and a poor prognosis. Currently, the gold standard for treating MIBC is radical cystectomy following cisplatin-based neoadjuvant chemotherapy (NAC) (77). However, two-thirds of MIBC patients exhibit partial or no pathological response to NAC, leading to delayed surgery and a worse prognosis (78). Therefore, accurately predicting the pathological response to NAC is crucial, as it significantly aids in the subsequent treatment and prognosis of MIBC patients (79). By employing whole exome sequencing (WES) to identify gene mutations in MIBC that can predict NAC response, Yang et al. discovered that BCa patients with mutations in METTL3 experienced a significant survival benefit after NAC treatment (80).

## 6 Potential clinical applications of METTL3-targeted therapeutic strategies in BCa

Currently, treatment strategies for BCa are rapidly evolving, particularly due to breakthroughs in immunotherapy (81, 82) and targeted therapy (83), which have significantly transformed the treatment landscape. Immune checkpoint inhibitors have been extensively utilized in the management of advanced or metastatic bladder cancer, demonstrating remarkable efficacy in prolonging patient survival and enhancing quality of life (84). Additionally, targeted therapies that focus on specific molecular characteristics provide tailored treatment options for patients with fibroblast growth factor receptor (FGFR) mutations or fusions (85). Furthermore, emerging therapies such as antibody-drug conjugates (ADCs) broaden the available treatment options (86). These advancements not only offer patients a wider array of choices but also advance bladder cancer treatment toward a more personalized and diverse approach. One such molecule, METTL3, is a key methyltransferase responsible for m^6^A modification and plays a crucial role in cancer development and progression. Targeting METTL3 as a therapeutic strategy for various types of tumors has received widespread attention (87). Compared to normal bladder tissues, METTL3 is highly expressed in BCa tissues and regulates multiple tumor characteristics, including cell proliferation, metastasis, anti-apoptosis, and chemoresistance. Therefore, targeting METTL3 may achieve multiple anti-tumor effects and possibly possess favorable tumor specificity. Currently, research on the development of METTL3-targeted therapies for BCa treatment is still mostly at an early stage, mainly focusing on small molecule inhibitors, RNA-targeted therapies, and combination treatment strategies.

### 6.1 Small molecule inhibitors

The development of small molecule inhibitors targeting METTL3 aims to inhibit its m^6^A methylation function and block its oncogenic effects in BCa (88). Although no METTL3-targeted drugs have been approved yet, inhibitors based on the structure of its methyltransferase, such as the METTL3/METTL14 heterodimer, are currently undergoing early-stage research. These inhibitors primarily fall into two categories: nucleoside and non-nucleoside compounds. STM2457 is the first widely studied small molecule inhibitor of METTL3. It competitively binds to the SAM binding site of METTL3, inhibiting its m^6^A methyltransferase activity and thereby reducing RNA methylation levels. In acute myeloid leukemia (AML) models, STM2457 significantly inhibits tumor cell proliferation, induces apoptosis, and blocks the proliferation and colony formation of the Human Acute Myeloid Leukemia Cell Line (MOLM-13), all without affecting normal hematopoietic function (89). STM2457 is also being considered for the treatment of non-small cell lung cancer (NSCLC), where it can upregulate programmed death-ligand 1 (PD-L1) both *in vivo* and *in vitro*, enhance the efficacy of NSCLC immunotherapy, and inhibit tumor progression while overcoming heterogeneity through its impact on the translatome (90). In addition to STM2457, several pharmaceutical companies and academic institutions are utilizing high-throughput screening technology to develop new small molecule inhibitors of METTL3, although these specific drugs are still in the early stages of research (91). A derivative of STM2457, known as STC-15, has reportedly entered clinical trials, and preliminary results indicate that it shows promise for inhibiting tumor growth through direct antitumor effects and anticancer immune responses (92). In summary, the successful development of STM2457 provides a crucial research foundation for targeting BCa treatment and holds significant potential in BCa therapy. By inhibiting m^6^A modification and blocking the stability and expression of oncogenic genes, it can effectively suppress the progression of BCa.

### 6.2 RNA-targeted therapy

Currently, there are few reports on RNA-targeted therapies for BCa; however, targeting METTL3 presents significant potential for BCa treatment, particularly in the realms of small interfering RNA (siRNA) (93)and antisense oligonucleotides (ASO) (94).

#### 6.2.1 SiRNA

siRNA technology can specifically silence the expression of target genes and has emerged as a promising treatment for cancer (95). By designing specific siRNAs to target the Mettl3 gene, it may be possible to inhibit the proliferation and invasion of BCa cells or enhance their chemosensitivity. Although there are few reports on siRNA targeting Mettl3 in BCa, the use of siRNA delivered by nanocarriers has become a new focus in cancer therapy (96). Studies have demonstrated that siRNA-mediated silencing of the METTL3 gene can significantly inhibit the proliferation and invasive capacity of BCa cells. *In vitro* experiments have shown that METTL3 knockdown leads to cell cycle arrest and increased apoptosis. In mouse models, siRNA-mediated silencing of the METTL3 gene significantly inhibits BCa tumor growth and metastasis (16, 64). However, the lack of effective *in vivo* delivery carriers remains a major challenge in translating siRNA into therapeutic drugs (97). Liu et al. found that utilizing natural halloysite nanotubes (HNTs) for nucleic acid delivery can address issues related to the low efficiency, rapid degradation, and toxicity of siRNA. HNT-encapsulated siRNA is more stable in serum, has a longer circulation time in the bloodstream, is more readily absorbed by BCa cells, and accumulates in BCa tumors (98). These findings provide valuable insights for the study of siRNA targeting Mettl3 in the treatment of BCa.

#### 6.2.2 ASO

ASOs are single-stranded DNA or RNA molecules that can bind to target mRNA through complementary base pairing, leading to mRNA degradation or the inhibition of translation (99). Li et al. discovered that in castration-resistant prostate cancer (CRPC), targeting METTL3 with ASO technology can significantly reduce the mRNA levels of METTL3, thereby diminishing its regulatory effects on downstream genes such as harvey rat sarcoma viral oncogene homolog (HRAS) and mitogen-activated protein kinase kinase 2 (MEK2), which in turn inhibits the proliferation and drug resistance of CRPC cells (100). Although there are currently no direct studies supporting the application of ASOs targeting METTL3 in BCa, research on ASOs for BCa treatment has consistently garnered significant interest (101). Given its molecular mechanism, the technological advancements in BCa treatment, and its applications in other tumors, this area warrants further investigation.

RNA-targeted therapies present an innovative approach for targeting METTL3 in BCa (88). Compared to traditional small molecule inhibitors, RNA-based therapies offer higher specificity and greater design flexibility, making them a promising therapeutic strategy (102). By utilizing technologies such as siRNA and ASO, the expression of METTL3 can be effectively reduced, its oncogenic effects can be inhibited, and consequently, the growth and metastasis of BCa can be suppressed. However, RNA-targeted therapies still encounter challenges related to delivery efficiency, off-target effects, and clinical translation (103). In the future, advancements in delivery technologies, chemical modifications, and precision medicine are expected to enhance the efficacy of RNA-targeted therapies, positioning them as a vital option for BCa treatment and providing patients with more precise and effective therapeutic solutions.

### 6.3 Combination treatment strategies

BCa is a complex disease associated with high incidence and mortality rates if not treated optimally. The primary treatment options include surgery, chemotherapy, radiotherapy, and immunotherapy; however, their effectiveness is often limited by patient-specific differences and drug resistance (2). In recent years, the oncogenic mechanisms of METTL3, a key enzyme involved in m^6^A methylation, have been extensively studied in BCa (104). Research has shown that METTL3 promotes tumor progression by stabilizing oncogenes, enhancing chemoresistance (17), and regulating the tumor immune microenvironment. Consequently, combination therapeutic strategies targeting METTL3 are emerging as a new research direction. Mao et al., by using the RNA Molecule Targeting (RM2Target) database, identified important regulatory associations between 20 pairs of prognostic immune genes (PIGs) and m^6^A regulators among the 28 PIGs identified. METTL3 and virulence factor protein (VIRMA) play key roles in immune-related m^6^A modifications, indicating that the design of inhibitors targeting METTL3 and VIRMA may represent a promising approach to combining anti-m^6^A therapy with immunotherapy (105). METTL3 significantly influences the function of immune cell subpopulations, including CD8+ T cells and myeloid-derived suppressor cells (MDSCs), by regulating the m6A modification of RNA. This regulation impacts the response to immunotherapy in BCa. In CD8+ T cells, METTL3 enhances the stability and translational efficiency of genes associated with the T cell receptor (TCR) signaling pathway, thereby promoting their proliferation, activation, and the expression of effector molecules, which ultimately strengthens anti-tumor immunity (106). Furthermore, METTL3’s regulation can decrease the expression of molecules related to T cell exhaustion, thereby improving the efficacy of immune checkpoint inhibitor therapies (107).Wang et al. found that by altering the tumor microenvironment and recruiting CD8+ tumor-infiltrating lymphocytes (TILs), inhibiting m^6^A modification can sensitize tumors to immunotherapy. The growth-inhibitory effects of Mettl3/14-deficient tumors are comparable to those of various combination immunotherapies, thus opening the door to combining immunotherapy with newly developed methyltransferase inhibitors for BCa treatment (106). In addition, Wu et al. found that inhibiting METTL3 can improve anti-programmed death protein 1 (PD-1) therapy in an m^6^A-YTHDF2-dependent manner. METTL3 inhibition or knockout affects tumor cell proliferation and tumor growth, with YTHDF2 playing a key role and enhancing antitumor effects in a T-cell-dependent manner, indicating that YTHDF2 is a downstream executor of STM2457’s antitumor effects (107). In MDSCs, METTL3 enhances the expression of inhibitory factors through m6A modification, thereby amplifying their immunosuppressive effects on T cells. Simultaneously, it regulates the metabolic pathways and differentiation of MDSCs, further bolstering the tumor’s capacity to evade the immune response (108). The latest studies have found that METTL3 increases C-X-C motif chemokine ligand 5 (CXCL5) levels and inhibits C-C motif chemokine ligand 5 (CCL5) expression in an m^6^A-dependent manner, leading to increased recruitment of MDSCs and reduced infiltration of CD8+ T cells. Silencing or inhibiting METTL3 can restore immune cell balance and significantly enhance the efficacy of anti-PD-1 therapy (109). These studies have identified METTL3 as a key regulator of the tumor immune microenvironment and a promising therapeutic target for improving immunotherapy outcomes. However, combination therapies targeting METTL3 still face challenges such as drug delivery efficiency, off-target effects, and toxicity (110), which need to be further optimized and confirmed for their safety and efficacy through clinical trials (111). These explorations provide new perspectives and research directions for the precision treatment of BCa.

## 7 METTL3 mediates drug resistance and poor prognosis in BCa

Chemotherapy is a crucial strategy for treating MIBC and metastatic BCa (112), particularly as adjuvant therapy before or after surgery or in cases where surgery is not feasible. Combination therapies, such as methotrexate, vinblastine, doxorubicin, and cisplatin (MVAC), as well as GC, are considered the primary treatment regimens for MIBC and metastatic BCa (113). However, the emergence of cisplatin resistance significantly limits therapeutic efficacy and adversely affects patient prognosis (114). METTL3 may become a potential biomarker for BCa resistance and prognosis assessment in BCa (18, 115), providing new targets for clinical treatment. It has been found that in BCa tissues and cell lines, a novel circRNA 0008399 (circ0008399), which is upregulated by the eukaryotic translation initiation factor 4A3 (EIF4A3), promotes the formation of the WTAP/METTL3/METTL14 m^6^A methyltransferase complex by binding to WTAP. It regulates the expression of target RNA through m^6^A modification and reduces cisplatin sensitivity and tumor occurrence and development in BCa (17). Meanwhile, Xu et al. found that the expression of circRNA 104797 (circ_104797) is upregulated in cisplatin-resistant BCa cells and plays a key role in maintaining cisplatin resistance. In addition, the demethylation of circ_104797 significantly enhances the efficacy of cisplatin-mediated apoptosis. Bioinformatics analysis also indicates potential interactions between circ_104797 and RNA-binding proteins (RBPs), and these findings suggest that METTL3-mediated m^6^A modification may regulate cisplatin resistance in BCa (116). The latest research has found that in cisplatin-resistant BCa cells, METTL3 stabilizes the mRNA of ring finger protein 220 (RNF220) through m^6^A modification, thereby promoting RNF220 protein expression. RNF220 can promote the ubiquitination and degradation of phosphodiesterase 10A (PDE10A), leading to a decrease in PDE10A protein levels and enhanced cisplatin resistance. At the same time, RNF220 can also destroy the stability of PDE10A and promote PD-L1 expression, leading to immune evasion. Therefore, METTL3 can indirectly affect PDE10A and PD-L1 through RNF220 to regulate drug resistance and immune evasion (117). Zhang et al. developed an m^6^A subtype classifier from the perspective of m^6^A, using single-sample gene set enrichment analysis(ssGSEA), estimation of STromal and immune cells in MAlignant tumors using expression data (ESTIMATE), microenvironment cell populations counter (MCPcounter), the tumor immune dysfunction and exclusion (TIDE) algorithm, Kaplan-Meier (K-M) survival curves, and cox proportional hazards model (Cox) regression analysis to identify patients with different prognostic risks and treatment responsiveness for precise treatment of BCa (118). It has also been shown that by investigating the copy number variation (CNV) status of 23 m^6^A methylation-related genes (MRGs) in the cancer genome atlas (TCGA) of BCa patients, 24.51% of the 411 TCGA BCa patients had mutations in these 23 genes, with METTL3 mutations being the most frequent, indicating that METTL3 is one of the key m^6^A MRGs in BCa and is related to BCa survival (119). Wang et al. used the TCGA database to study CNVs of all known m^6^A regulatory genes and found that CNVs of METTL3, METTL14, and METTL16 are associated with the molecular characteristics of BCa patients, and CNVs of METTL3 are also associated with the overall survival (OS) of BCa patients. Therefore, METTL3 is a prognostic and immune-related biomarker for BCa (120).Yan et al. discovered that melittin can selectively induce apoptosis in BCa cells through a METTL3-dependent mechanism. METTL3 facilitates the maturation of primary microRNA-146 (pri-miR-146) via m^6^A modification, while microRNA-146a-5p (miR-146a-5p) exerts oncogenic effects by regulating the NUMB protein and NOTCH2 receptor (NUMB/NOTCH2) axis. Inhibiting METTL3 or miR-146a-5p can enhance the antitumor effects of melittin; thus, high expression levels of METTL3 and miR-146a-5p are associated with BCa recurrence and poor prognosis (121). Overall, targeting METTL3 may represent a promising therapeutic strategy to overcome resistance in BCa and improve patient outcomes.

## 8 Discussion

BCa is a highly heterogeneous malignant tumor characterized by a complex pathogenesis, and its treatment outcomes are often hindered by chemoresistance and high recurrence rates. In recent years, epigenetic research has increasingly highlighted the significant role of m^6^A RNA methylation in tumor initiation and progression (122). As a key enzyme responsible for m^6^A modification, METTL3 has attracted considerable attention for its functions across various cancer types. In BCa, METTL3 exhibits multifaceted oncogenic roles, significantly influencing patient prognosis by promoting tumor cell proliferation, migration, invasion, and chemoresistance. Studies have demonstrated that METTL3 enhances tumor cell proliferation and invasion through the MYC, inhibitor of nuclear factor kappa-B kinase subunit beta (IKBKB), and RELA proto-oncogene, NF-kB subunit (RELA) signaling pathways. Furthermore, METTL3 is closely associated with chemoresistance in BCa (116) and poor patient outcomes (119). These findings suggest that METTL3 not only serves as a potential therapeutic target for BCa but also possesses clinical value as a prognostic biomarker. Although the oncogenic roles of METTL3 in BCa have been extensively documented, its specific molecular mechanisms warrant further investigation. The interactions of METTL3 with other m^6^A regulators, such as FTO and ALKBH5, in either a synergistic or antagonistic manner remain largely unexplored. While therapeutic strategies targeting METTL3 present promising prospects for BCa treatment, In the context of combination therapy strategies for BCa, the role of METTL3 in ADC therapy, as well as ADC combined with immunotherapy, requires further in-depth exploration. A multicenter, real-world cohort study conducted by Hu et al. included 253 patients receiving neoadjuvant treatment across 15 tertiary hospitals (98 patients in combination therapy, 107 in chemotherapy, and 48 in immunotherapy). The results indicated that neoadjuvant combination therapy significantly outperformed single-agent chemotherapy or immunotherapy, achieving the highest rates of complete response and pathological downstaging. This finding underscores the clear advantage of combination therapy in enhancing patient prognosis (123). Furthermore, their latest research confirmed that Disitamab Vedotin (RC48-ADC) combined with immunotherapy exhibited good efficacy in patients with MIBC who were not suitable for cisplatin. However, the durability of this efficacy and its safety still need to be validated through longer follow-up studies. Additionally, RC48-ADC is currently primarily utilized domestically, and its global application faces certain challenges, highlighting the importance of future international collaborative studies (124). Currently, no specific drugs have been clinically approved for the treatment of BCa, and several challenges hinder the development of METTL3-targeting drugs. Firstly, issues such as the delivery efficiency of targeted therapies, off-target effects, potential toxic side effects, low bioavailability, and insufficient specificity limit their practical application in BCa treatment. Secondly, the limitations of existing research data—including inadequate sample sizes, heterogeneity in experimental design, and inconsistencies in results—result in a lack of comprehensive understanding of METTL3’s mechanisms. Additionally, there are challenges with the research methodologies themselves, such as limitations in the sensitivity and specificity of m6A modification detection techniques, which compromise the reliability of the findings. Therefore, to effectively translate METTL3 from basic research to clinical application, continuous improvements are necessary in drug development, data accumulation, and methodological optimization. Nevertheless, therapeutic strategies aimed at METTL3 offer new avenues for precision treatment of BCa, although their clinical translation necessitates more comprehensive research.

## 9 Conclusions and future prospects

In recent years, research on m^6^A RNA methylation has deepened, gradually revealing the role of METTL3, a key enzyme for methylation, in BCa has gradually been revealed. Existing studies indicate that METTL3 promotes the proliferation, migration, invasion, and chemoresistance of BCa cells by regulating the stability and translation efficiency of oncogenes and tumor suppressor genes through m^6^A modification. Additionally, it facilitates immune evasion by modulating the tumor immune microenvironment. These functions position METTL3 as a significant potential target for BCa treatment and a crucial molecular marker for predicting patient prognosis. Although significant progress has been made in understanding the oncogenic role and molecular mechanisms of METTL3, many questions remain to be explored. First, how METTL3 collaborates with or opposes other m^6^A regulators in BCa and the specific networks and mechanisms of its action are still unclear. whether there are differences in the expression characteristics and functional roles of METTL3 in different BCa subtypes requires further investigation. Secondly, the current accumulation of research data and the existing methodological limitations require further enhancement. Additionally, therapeutic strategies targeting METTL3 remain in the exploratory phase. The development of highly specific, low-toxicity targeted drugs, along with the assessment of their combined effects with existing treatment modalities, necessitates further validation through preclinical and clinical studies. However, the advancement of METTL3-targeted drugs encounters significant challenges, including low bioavailability, off-target effects, and the absence of efficient delivery systems. Moreover, the limited sample sizes and heterogeneity in the experimental designs of current studies restrict the broader applicability of the findings. Addressing these issues will establish a solid foundation for the clinical application of METTL3 in bladder cancer. With the continuous advancement of RNA epigenetic tools and technologies, therapeutic strategies targeting METTL3 are anticipated to be clinically translated in BCa. By integrating multi-omics data, including genomics, transcriptomics, and epigenetics, the precise role of METTL3 in BCa progression can be further elucidated, providing a foundation for the development of personalized treatment plans. Furthermore, as a prognostic marker for BCa, the clinical diagnostic value of METTL3 requires additional validation, particularly in predicting disease recurrence, metastatic risk, and sensitivity to chemotherapy and immunotherapy. Overall, therapeutic strategies targeting METTL3 not only have the potential to overcome chemoresistance in BCa but may also enhance the efficacy of existing immunotherapy outcomes, thereby improving overall survival rates and quality of life for patients. In the future, a comprehensive exploration of the molecular mechanisms of METTL3 and the promotion of its clinical application will yield more precise and effective solutions for the diagnosis and treatment of BCa.
